# 

**Published:** 1907-06-15

**Authors:** 


### ANNOUNCEMENTS.

The Virginia State Dental Association will hold its Annual Meeting the 9th of September, 1907, at the Inside Inn, Jamestown Exposition. There will only be a short session, as the activities of our members are being merged with those of the Jamestown Dental Convention. This will be strictly a business meeting. No committees will be appointed, and no work done other than certain important matters of business, which will be designated later in a circular letter to be issued to each member.—

W. H. Pearson,
*Asst. Cor. Secty.*

National Dental Association Program—Section 1. —The following papers have been secured for Section 1 of the National Dental Association, for the Minneapolis meet ing, beginning July 30th:

1st, "The Over-Arch Bar in Bridgework," Dr. L. C. Bryan, Bazel, Switzerland; 2nd, "Some Practical Experiences Theoretically Expressed," Dr. Emory A. Bryant, Washington, D. C.; 3rd, "Treatment of Mal-Occlusion of Deciduous Teeth." Dr. Guilhermena P. Mendel, Minneapolis, Minn.; 4th "Evolution," Dr. Chas. L. Hungerford, Kansas City, Mo.; 5th, "The Effect of Excess of Mercury upon Shrinkage, Expansion, Edge Strength, Flow, Change in Composition and stability of the Dental Amalgam Alloys," Dr. Marcus L. Ward, Detroit, Mich.; 6th, "Porcelain," Dr. C. M. Work, Ottumwa, Iowa; 7th, "Physical Condition of, or Pertaining to the Human Teeth," Dr. F. G. Corey, Council Groves, Kans.; 8th, "Method of Replacing Broken Facings on Crowns and Bridges," Dr.. V. Conzett, Dubuque, Iowa.

There may be a few additions to the list, as all the returns are not yet in.

D. M. LeCron, *Chmn. Sec.* 1, *N. D. A.*
E. P. Dameron, *Secy. Sec.* 1, *N. D. A.*

—✦—

The Wyoming State Board of Dental Examiners. —Will meet at Sheridan, Wyo., on July 1, 2, and 3, 1907, or the examination of candidates. All applications must be in the hands of the secretary not later than June 25th.

For further information and application blanks, address

Peter Appel, Jr., *Secretary,*

P. O. Box 643, Cheyenne, Wyo.

OHIO STATE BOARD OF DENTAL EXAMINERS.—The regular semi-annual meeting of the Board of Dental Examiners of the State of Ohio will be held in Columbus, June 25, 26, and 27, 1907. Only graduates are eligible to examination.

Application, accompanied by fee ($20) should be filed with the secretary by June 15. For further information address:

H. C. BROWN, *Secretary*,
185 East State Street, Columbus, Ohio.

REPORT OF THE SAN FRANCISCO DENTAL RELIEF COMMITTEE.—This committee sends out a very comprehensive report of its work in connection with relieving distressed members of the profession and their families during the earthquake disaster. The treasurer's report is as follows:

| | | |
|---|---|---|
| Receipts from April 28, 1906 to December 30, 1906 | | $15,208.45 |
| Disbursements: | | |
| Relief orders | $10,783.75 | |
| Newspaper announcements | 65.40 | |
| Mimeograph Work | 19.25 | |
| Postage | 22.75 | |
| Clerical service | 315.00 | |
| Freight | 32.55 | $11,238.70 |
| Balance on hand | | $ 3,969.75 |

It is proposed by the committee to turn the balance on hand over to the National Dental Association as a nucleus for a National relief fund, and it is suggested that the profession increase this fund until it reaches $50,000, for the purpose of taking care of worthy members of the profession who may come to need in their declining years.

---

THE AMERICAN DENTAL SOCIETY OF EUROPE.—List of officers for the ensuing year: President, Dr. Kirk Davenport, London; Vice-president, Dr. Charles Rathbun, London; Treasurer, Dr. William M. Cooper, Frankfort; Secretary, Dr. J. W. Gale, Cologne; The next meeting will be held in London in August, 1908.

J. W. GALE, *Hon. Secretary.*

